# Direct conversion of human fibroblasts to brown adipocytes by small chemical compounds

**DOI:** 10.1038/s41598-017-04665-x

**Published:** 2017-06-27

**Authors:** Yukimasa Takeda, Yoshinori Harada, Toshikazu Yoshikawa, Ping Dai

**Affiliations:** 1 Department of Cellular Regenerative Medicine, Graduate School of Medical Science, Kyoto Prefectural University of Medicine, 465 Kajii-cho, Kawaramachi-Hirokoji, Kamigyo-ku, Kyoto, 602-8566 Japan; 2 Department of Pathology and Cell Regulation, Graduate School of Medical Science, Kyoto Prefectural University of Medicine, 465 Kajii-cho, Kawaramachi-Hirokoji, Kamigyo-ku, Kyoto, 602-8566 Japan

## Abstract

Brown adipocytes play an important role in human energy metabolism and prevention of obesity and diabetes. Induced pluripotent stem cells (iPSCs) represent a promising source for brown adipocytes; however, exogenous gene induction is generally required for iPSCs generation, which might cause undesired effects particularly in long-term treatment after transplantation. We have previously reported a cocktail of six small chemical compounds that enables a conversion of human fibroblasts into chemical compound-induced neuronal cells (CiNCs). Here, we report that modified combinations of the chemical compounds and rosiglitazone, a PPARγ agonist, afforded direct conversion of human fibroblasts into brown adipocytes. The chemical compound-induced brown adipocytes (ciBAs) exhibit induction of human brown adipocyte-specific genes such as *Ucp1*, *Ckmt1*, *Cited1* and other adipocyte-specific genes such as *Fabp4*, *AdipoQ*, and *Pparγ*. Treatment with either isoproterenol or Forskolin further induced the expression of *Ucp1*, suggesting that β adrenergic receptor signalling in ciBAs could be functional for induction of thermogenic genes. Moreover, oxygen consumption rates were elevated in ciBAs along with increase of cellular mitochondria. Our findings might provide an easily accessible approach for generating human brown adipocytes from fibroblasts and offer therapeutic potential for the management of obesity, diabetes, and related metabolic disorders.

## Introduction

Cell plasticity confers flexibility to change the fate of terminally differentiated cells into a pluripotent state or other cell types^1, 2^. Induced pluripotent stem cells (iPSCs) are pivotal resources to provide desired cell types, but the generation of iPSCs possibly entails unexpected incorporation and mutation in genomic DNA due to transfection of exogenous DNA delivered by viruses or plasmids, which might cause undesired effects after transplantation^3^. Moreover, the differentiation capacity of each iPSC colony is heterogenous, and the process of selecting colonies is very costly and time-consuming. In this regard, direct lineage reprogramming is a promising alternative concept to prepare desired cell types rapidly without going through a pluripotent state. As already reported, human dermal fibroblasts can be directly converted into myoblasts, cardiomyocytes, hepatocytes, and neurons^4–6^, but overexpression of a set of transcription factors by means of transfection of exogenous DNA is required to establish similar transcriptional network, which makes application of directly reprogrammed cells for human regenerative medicine difficult.

Recently, regulation of cellular signalling pathways by small molecules has been developed to modulate cellular differentiation and reprogramming, which might be safer and replaced with gene inductions^4, 7–9^. Direct reprogramming using small compounds is one of the most ideal methods to prepare target cells that can be applied for rapid and more secure transplantation therapy. However, a limited number of human cell types such as neurons, cardiomyocytes, and astrocytes has been converted with small compounds so far^10–13^. Therefore, direct reprogramming to other target cell types by small compounds is an anticipated step to accelerate development of cell transplantation therapy.

We have previously reported that efficient direct conversion of human fibroblasts into neural cells can be achieved by using a combination of six chemical compounds and the neuronal medium^14^. This finding encouraged us to try the combination of these small compounds with various culture media to develop more numerous target cell types from human fibroblasts. In this study, we treated human fibroblasts with the six chemical compounds in combination with a commercial adipocyte medium. We successfully converted chemical compound-induced brown adipocytes (ciBAs) from human fibroblasts. The ciBAs might have a therapeutic potential for the treatment of human metabolic diseases.

## Results

### Identification of chemical compounds for direct conversion from human fibroblasts to adipocyte-like cells

**Figure 1:**
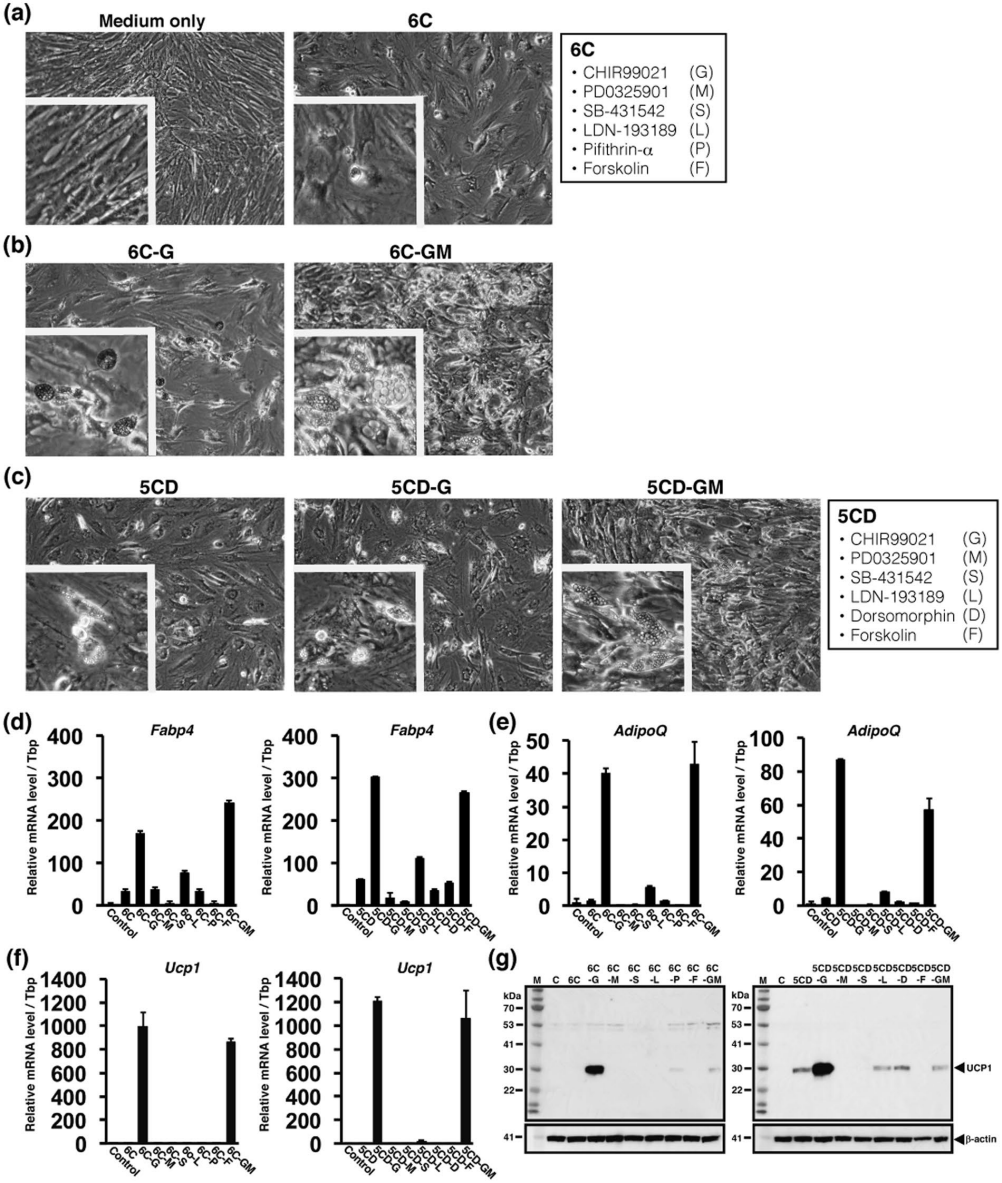
Identification of chemical compounds for direct conversion from HDF38 fibroblasts to adipocyte-like cells. Effects of chemical compounds were evaluated 3 weeks after the treatment. (**a**) Images after the treatment using the adipocyte medium only or the adipocyte medium supplemented with the 6 chemical compounds (6C). Insets show higher magnification. (**b**) Morphologies after cultivation with the adipocyte medium and the chemical cocktail excluding either CHIR99021 (G) or CHIR99021 and PD0325901 (GM) from 6 C. Insets show adipocyte-like cells. (**c**) Images after treatment with the adipocyte medium with 5 CD (5C supplemented with Dorsomorphin (D)) (left panel), and the adipocyte medium with chemical cocktails excluding either G (middle panel) or GM (right panel) from 5CD. Insets show adipocyte-like cells. qRT-PCR analyses of adipocyte-specific genes, *Fabp4* (**d**) and *AdipoQ* (**e**), and a brown adipocyte-specific gene, *Ucp1* (**f**), after cultivation with the adipocyte medium only (Control) and the medium supplemented with each combination of the chemical compounds as indicated. (**g**) UCP1 protein levels were evaluated by western blotting analysis. HDF38 fibroblasts were cultured as described above. β-actin is a loading control. “M” and “C” indicate a molecular weight marker and the control, respectively. Data represent mean ± SD.

Fibroblasts derived from a human subject at age 38 yrs (HDF38) (Table 1) were cultured with different types of culture media for 3 weeks to identify chemical compounds for direct conversion to adipocyte-like cells. Firstly, HDF38 was incubated with the adipocyte medium including rosiglitazone. Rosiglitazone is reported to play a critical role in adipogenesis^15^. Treatment with the adipocyte medium only did not induce a dramatic change of morphology of the fibroblasts (left panel of Fig. 1a). However, a small amount of cells with lipid droplets appeared 3 weeks after the incubation with the adipocyte medium in combination with the 6 chemical compounds (6 C) (right panel of Fig. 1a) which consists of a GSK3β inhibitor, CHIR99021 (G), a MEK inhibitor, PD0325901 (M), a TGF-β signalling inhibitor, SB-431542 (S), a BMP signalling inhibitor, LDN-193189 (L), a P53 inhibitor, Pifithrin-α (P), and a cAMP inducer, Forskolin (F). When CHIR99021 was excluded from the 6 C (6C-G), more cells with clear lipid droplets were generated (left panel of Fig. 1b). 6C-GM, in which CHIR99021 and PD0325901 were excluded from the 6 C, resulted in formation of better adipocyte-like cells with larger lipid droplets, which were similar to typical adipocytes (right panel of Fig. 1b).

**Table 1:** Information on human fibroblasts used for direct conversion.

Abbreviation	Lot#	Passage	BMI	Age	Gender	Site
HDF0	DFMF112410B	4	Unknown	0	Male	Foreskin
HDF38	DFM090214A	3	23.1	38	Male	Abdomen
HDF49	3101601.2	2	Unknown	49	Female	Breast

Since P53 is critical for maintenance of genomic DNA^16^, we prepared 5 C cocktail containing CHIR99021, PD0325901, SB-431542, LDN-193189, and Forskolin by removing Pifithrin-α from the 6 C. The removal of Pifithrin-α did not affect efficiency of the direct conversion into adipocyte-like cells (data not shown). In addition, we found that addition of Dorsomorphin (D), an inhibitor of BMP signalling pathway and AMPK, to the 5 C promoted the direct conversion (left panel of Fig. 1c). Similar to the observations with the combination of 6 C, the removal of CHIR99021 or both CHIR99021 and PD0325901 from the new combination, 5CD, improved the conversion efficiency (middle and right panels of Fig. 1c). Therefore, Dorsomorphin was utilized instead of Pifithrin-α for subsequent experiments.

In order to support the observations described above, expression of several adipocyte-specific genes was quantified in the cells treated with each combination of chemical compounds. As shown in Fig. 1d, *Fabp4* expression was slightly increased in the treatment with the 6 C compared with the control treated with the adipocyte medium only. The expression was further induced in both 6C-G and 6C-GM, consistent with the observation that adipocyte-like cells were more abundantly generated by the removal of G. 5CD-G and 5CD-GM also induced *Fabp4* expression at a higher rate than the 6 C combination. The expression of another adipocyte-specific gene, *AdipoQ*, was also particularly increased by excluding G and GM from the combinations, 6 C and 5CD (Fig. 1e). Since lipid droplets were not large enough compared to typical white adipocytes, we also tested the expression of *Ucp1*, a hallmark of brown adipocytes. The expression of *Ucp1* mRNA (Fig. 1f) and UCP1 protein (Fig. 1g) was induced by removing G and GM from 6 C and 5CD, suggesting that chemically induced adipocyte-like cells might have a feature of brown adipocytes rather than white adipocytes.

Next, we thoroughly tested combinations of the small compounds to find the optimum one for the direct conversion into brown adipocyte-like cells (Figs S1–S5). After incubation of HDF38 fibroblasts with each chemical cocktail for 3 weeks, HDF38 continued to be cultured for another 1 week with the adipocyte medium without the chemicals to promote maturation of brown adipocyte-like cells. As shown in Fig. S1, 5CD-G efficiently generated the brown adipocyte-like cells expressing UCP1. Then, one of the chemicals was further removed from the combination of 5CD-G (Fig. S2). The additional removal of either M or L resulted in the most efficient generation of the brown adipocyte-like cells. However, 5CD-GL underwent a small amount of cell death during the conversion, so we determined that the combination of 5CD-GM containing SB-431542 at 2 μM, LDN-193189 at 1 μM, Dorsomorphin at 1 μM, and Forskolin at 7.5 μM was the optimum one to generate brown adipocyte-like cells with the smallest rate of cell death (Table 2).

**Table 2:** Efficiency of direct conversion into brown adipocyte-like cells by each chemical cocktail.

Combination	Efficiency	Note
**6 compounds**		
5CD (GMSLDF)	+	
**5 compounds**		
5CD-G (MSLDF)	+++	
5CD-M (GSLDF)	+	
5CD-S (GMLDF)	+	
5CD-L (GMSDF)	++	
5CD-D (GMSLF)	+	
5CD-F (GMSLD)	+	Cell death
**4 compounds**		
5CD-GM (SLDF)	++++	
5CD-GS (MLDF)	+	Cell death
5CD-GL (MSDF)	++++	Cell death
5CD-GD (MSLF)	+++	Cell death
5CD-GF (MSLD)	++	
**3 compounds**		
MSL	++	
MSD	+++	
MSF	++	Cell death
MLD	++	
MLF	+	Cell death
MDF	++	Cell death
SLD	+++	
SLF	+++	
SDF	+++	
LDF	++	
**2 compounds**		
MS	++	
ML	−	
MD	++	
MF	++	
SL	+++	
SD	++	
SF	+++	
LD	+	
LF	++	
DF	+	
**1 compound**		
G	−	
M	+	
L	+	
S	++	
D	+	
F	+	Cell death
P	−	
Medium only	−	

Rosiglitazone was included in the adipocyte medium. HDF38 fibroblasts were utilized to evaluate the efficiency.

The combinations of 3 chemicals or fewer for direct conversion into brown adipocyte-like cells were also examined as shown in Figs S3, S4 and S5. The combinations including 3 chemicals such as MSD, SLD, SLF, and SDF provided relatively efficient conversion to the brown adipocyte-like cells (Fig. S3). The combination of two chemicals or a single chemical did not show a better efficiency than 5CD-GM (Figs S4 and S5); however, these results suggested that SB-431542, an inhibitor of TGF-β signalling pathway, plays a particularly important role in the direct conversion.

### Ucp1 expression and increased mitochondria biogenesis in chemically induced- brown adipocyte-like cells

**Figure 2:**
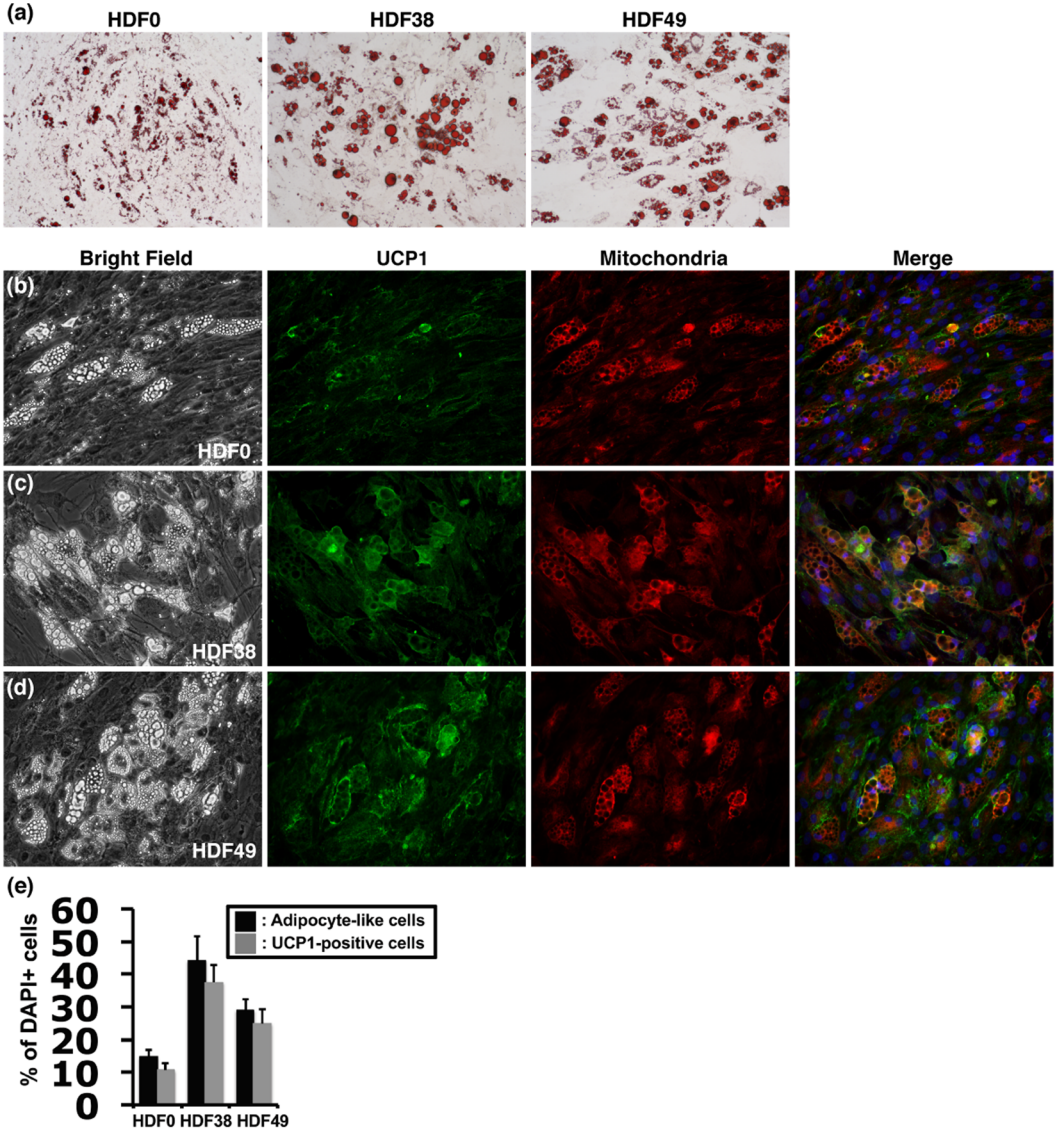
Chemically induced brown adipocyte-like cells exhibit a high abundance of lipid droplets and UCP1 expression along with elevated level of cellular mitochondria. The cells were induced by treatment with the adipocyte medium and 5CD-GM for 3 weeks followed by cultivation with the adipocyte medium only for 1 week. (**a**) Oil Red O staining of the adipocyte-like cells derived from the 3 lines of human fibroblasts, HDF0 (left panel), HDF38 (middle panel), and HDF49 (right panel). Images of bright field, UCP1 protein expression (green), mitochondria labelling with MitoTracker (red), and merged image of UCP1 (green), MitoTracker (red) and DAPI (blue) to visualise nuclei in the fibroblasts of HDF0 (**b**), HDF38 (**c**), and HDF49 (**d**). (**e**) To evaluate the efficiency of direct conversion by 5CD-GM, the numbers of DAPI-positive cells, adipocyte-like cells with lipid droplets, and UCP1-positive cells were counted in the 3 lines of human fibroblasts, HDF0, HDF38, and HDF49 (n = 3). Data represent mean ± SD.

Next, to confirm availability of the chemical cocktail, 5CD-GM, for the direct conversion, 2 other lines of human fibroblasts (HDF0 and HDF49) in addition to HDF38 (Table 1) were examined. After incubation with the adipocyte medium and 5CD-GM for 3 weeks, each fibroblast line was cultured for another 1 week with the adipocyte medium without 5CD-GM for maturation. All of the fibroblasts successfully generated the adipocyte-like cells (Fig. 2a and leftmost panels of Fig. 2b,c,d). Oil Red O staining confirmed that adipocyte-like cells had abundant lipid droplets (Fig. 2a). However, the conversion efficiency and Oil Red O staining seemed to be relatively lesser in HDF0 fibroblasts compared to the other fibroblasts, HDF38 and HDF49.

Immunocytochemical analysis was also performed to analyse protein expression and cellular localisation of UCP1 (left middle panels of Fig. 2b,c,d). The results revealed that most of the adipocyte-like cells were UCP1 positive, suggesting that they have a feature of brown adipocytes. In addition, UCP1 staining was well overlapped with elevated mitochondria signals (right middle and rightmost panels of Fig. 2b,c,d). The conversion efficiency into the adipocyte-like cells with lipid droplets and UCP1-positive cells by the treatment with 5CD-GM combination was around 10%, 40%, and 25% in HDF0, HDF38, and HDF49 fibroblasts, respectively (Fig. 2e). Hereafter, we designated the brown adipocyte-like cells as ciBAs, chemical compound-induced brown adipocytes.

In order to clarify whether ciBAs are maintained for a long time without the chemical compounds, they continued to be cultured with the adipocyte medium only after treatment with 5CD-GM for the first 3 weeks. At Day 60, the morphology of the adipocyte-like cells with lipid droplets was maintained in ciBAs derived from HDF38 and HDF49 fibroblasts (Fig. S6a,b). Moreover, mature adipocyte-like cells appeared to be rather increased at Day 60 compared to the ones at Day 30. Immunocytochemical analysis in ciBAs at Day 60 indicated that UCP1 expression was maintained and overlapped with elevated mitochondria signals, which is similar to the ones at Day 28 in Fig. 2c. These results suggest that ciBAs are stable and maintained over 2 months without the chemical compounds for the direct conversion in this study.

### Induced expression of human brown adipocyte-specific genes in ciBAs

**Figure 3:**
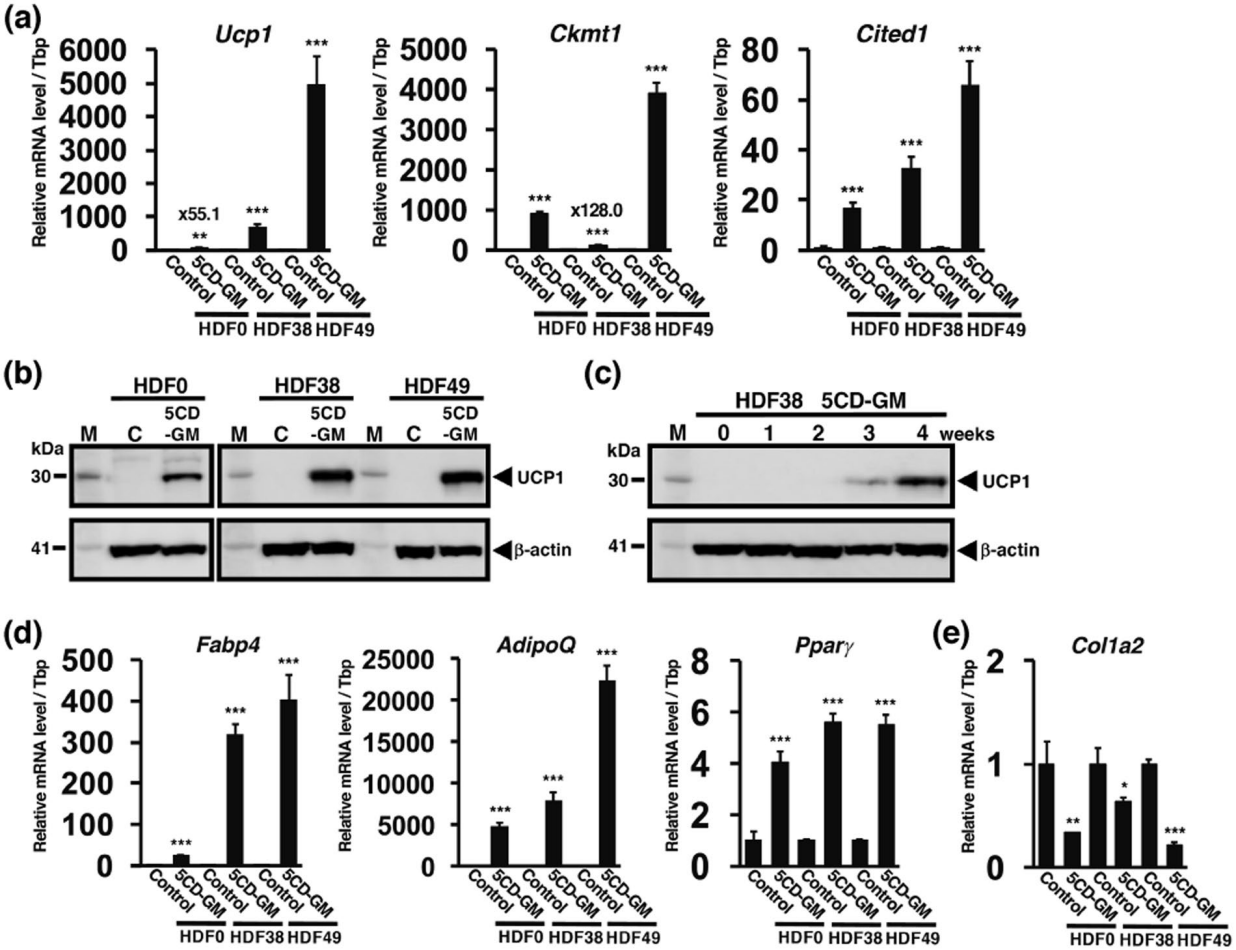
Human brown adipocyte-specific gene expression in chemical compound-induced brown adipocytes (ciBAs). ciBAs were induced by treatment with the adipocyte medium and 5CD-GM for 3 weeks followed by cultivation with the adipocyte medium only for 1 week. (**a**) qRT-PCR analyses of human brown adipocyte-specific genes, *Ucp1*, *Ckmt1*, and *Cited1* in the control cells (the adipocyte medium only) and ciBAs derived from the 3 lines of human fibroblasts, HDF0, HDF38, and HDF49. (**b**) The protein expression of UCP1 was evaluated by western blotting analysis in ciBAs derived from the 3 lines of human fibroblasts. (**c**) UCP1 protein levels in HDF38 fibroblasts were evaluated every 1 week after treatment with 5CD-GM. The cells during the period from 3 to 4 weeks were cultured with the adipocyte medium only for the maturation. β-actin is a loading control. “M” and “C” indicate a molecular weight marker and the control, respectively. (**d**) qRT-PCR analyses of *Fabp4*, *AdipoQ*, and *Pparγ* that are preferentially expressed in adipocytes. (**e**) qRT-PCR assay of a fibroblast-specific gene *Col1a2*. Data represent mean ± SD (n = 3). Student’s t-test: *P < 0.05, **P < 0.01, ***P < 0.001.

To characterize gene expression of ciBAs, several marker genes specific for human brown adipose tissue (BAT) were quantified by quantitative reverse transcriptase polymerase chain reaction (qRT-PCR). The 3 lines of the human fibroblasts, HDF0, HDF38, and HDF49, were cultured with the adipocyte medium and 5CD-GM for 3 weeks, followed by the maturation with the medium only for 1 week. Figure 3a indicates that *Ucp1* mRNA was induced compared to each control cultured with the medium only in all of the examined fibroblasts. Consistent with the observation shown in Fig. 2a, HDF0 fibroblasts were less efficiently converted into ciBAs than the other fibroblasts, HDF38 and HDF49. The expression of *Ckmt1*, mitochondrial creatine kinase^17^, and *Cited1*, a human brown adipocyte marker^18^, was induced in all the fibroblasts. The protein expression of UCP1 was also induced in ciBAs derived from all the fibroblast lines (Fig. 3b). In addition, we confirmed that UCP1 protein was expressed 3 weeks after the treatment with 5CD-GM and more enhanced by the cultivation for 1 week with the adipocyte medium only for the maturation (Fig. 3c). ciBAs exhibited increased expression of adipogenic differentiation markers, *Fabp4*, *AdipoQ*, and *Pparγ*, in all the fibroblasts (Fig. 3d). *Col1a2*, a fibroblast marker gene, was reduced about 40% to 80%, suggesting that the cell fate might be changed from the fibroblasts to the brown adipocyte-like cells or intermediate cells (Fig. 3e). These results suggest that ciBAs converted from the fibroblasts exhibited gene expression specific to that of human brown adipocytes.

### Functional β adrenergic receptor signalling for thermogenesis in ciBAs

**Figure 4:**
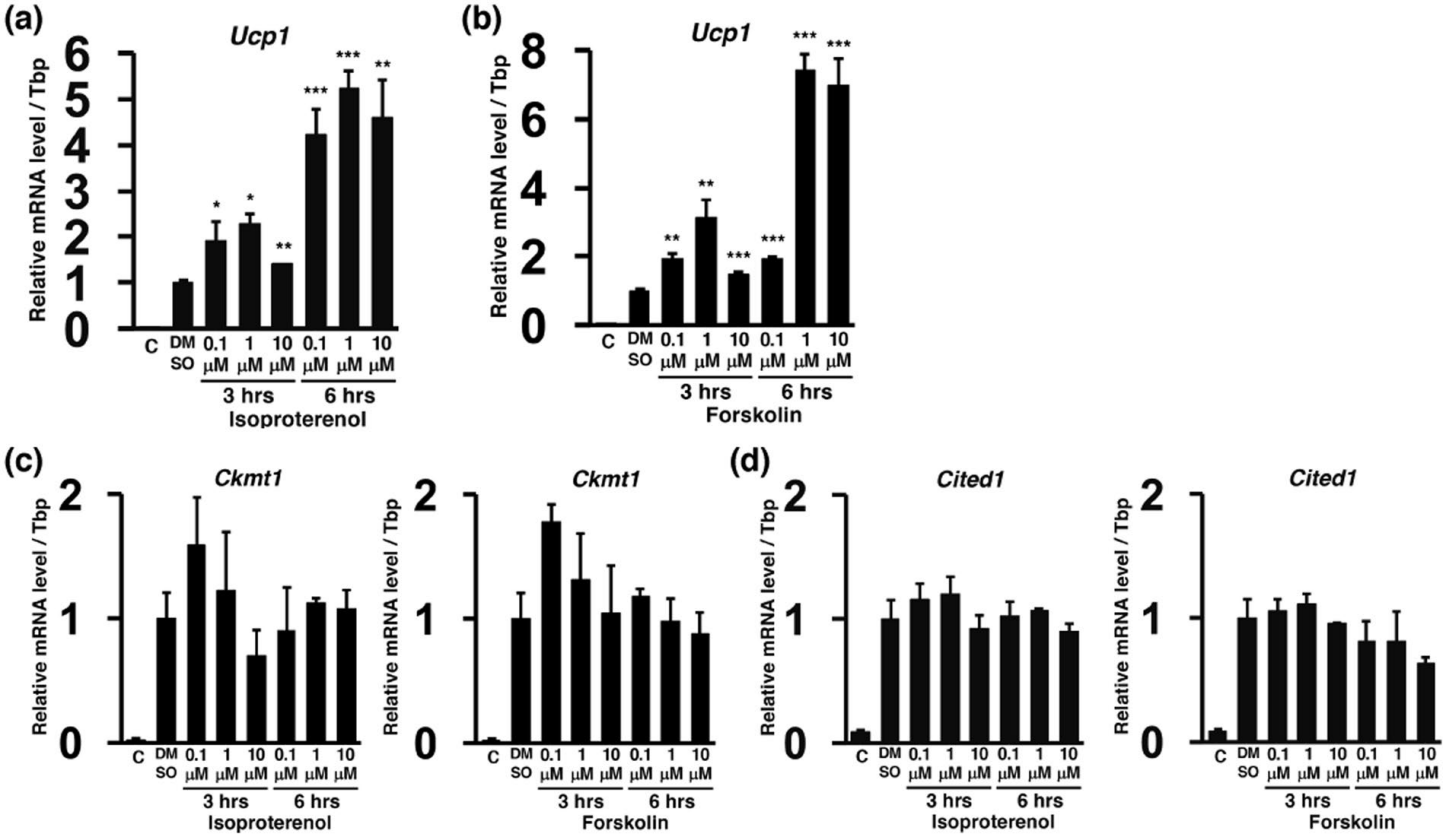
Induced expression of *Ucp1* mRNA by isoproterenol and Forskolin in ciBAs. qRT-PCR analyses of *Ucp1* mRNA in ciBAs converted from HDF38 fibroblasts after treatment with either isoproterenol (**a**) (0.1, 1, and 10 μM) or Forskolin (**b**) (0.1, 1, and 10 μM) for 3 hrs and 6 hrs as indicated. The expression level of ciBAs treated with dimethyl sulfoxide (DMSO) was used to normalize the expression levels. “C” at the first lane represents the expression level of HDF38 fibroblasts treated with the adipocyte medium only. The expression of other brown adipocyte-specific genes, *Ckmt1* (**c**) and *Cited1* (**d**), was quantified in the same manner as described above. Data represent mean ± SD (n = 3). Student’s t-test: *P < 0.05, **P < 0.01, ***P < 0.001.

Thermogenesis through *Ucp1* is regulated by the sympathetic nervous system and β adrenergic receptor signalling pathways in brown adipocytes. In order to evaluate thermogenic capacity in ciBAs, *Ucp1* mRNA was quantified after treatment with isoproterenol, an agonist for β adrenergic receptors, for 3 hrs and 6 hrs at 3 different concentrations (Fig. 4a). The treatment for 3 hrs slightly enhanced *Ucp1* mRNA, and 6 hrs treatment significantly enhanced about 4- to 5- fold compared to DMSO-treated ciBAs. To address whether the *Ucp1* induction is dependent on increased concentration of cellular cAMP, Forskolin was applied in ciBAs for 3 hrs and 6 hrs at 3 different concentrations in the same manner (Fig. 4b). *Ucp1* expression was more enhanced in ciBAs treated for 6 hrs than for 3 hrs compared to DMSO-treated ciBAs. In contrast, the expression of other human brown adipocyte specific genes, *Ckmt1* and *Cited1*, was not enhanced, implying that the upregulation might be specific to *Ucp1* gene (Fig. 4c,d). These results suggest that ciBAs might be responsive to β adrenergic receptor signalling pathways for thermogenic gene induction.

### Elevation of oxygen consumption rate (OCR) in ciBAs

**Figure 5:**
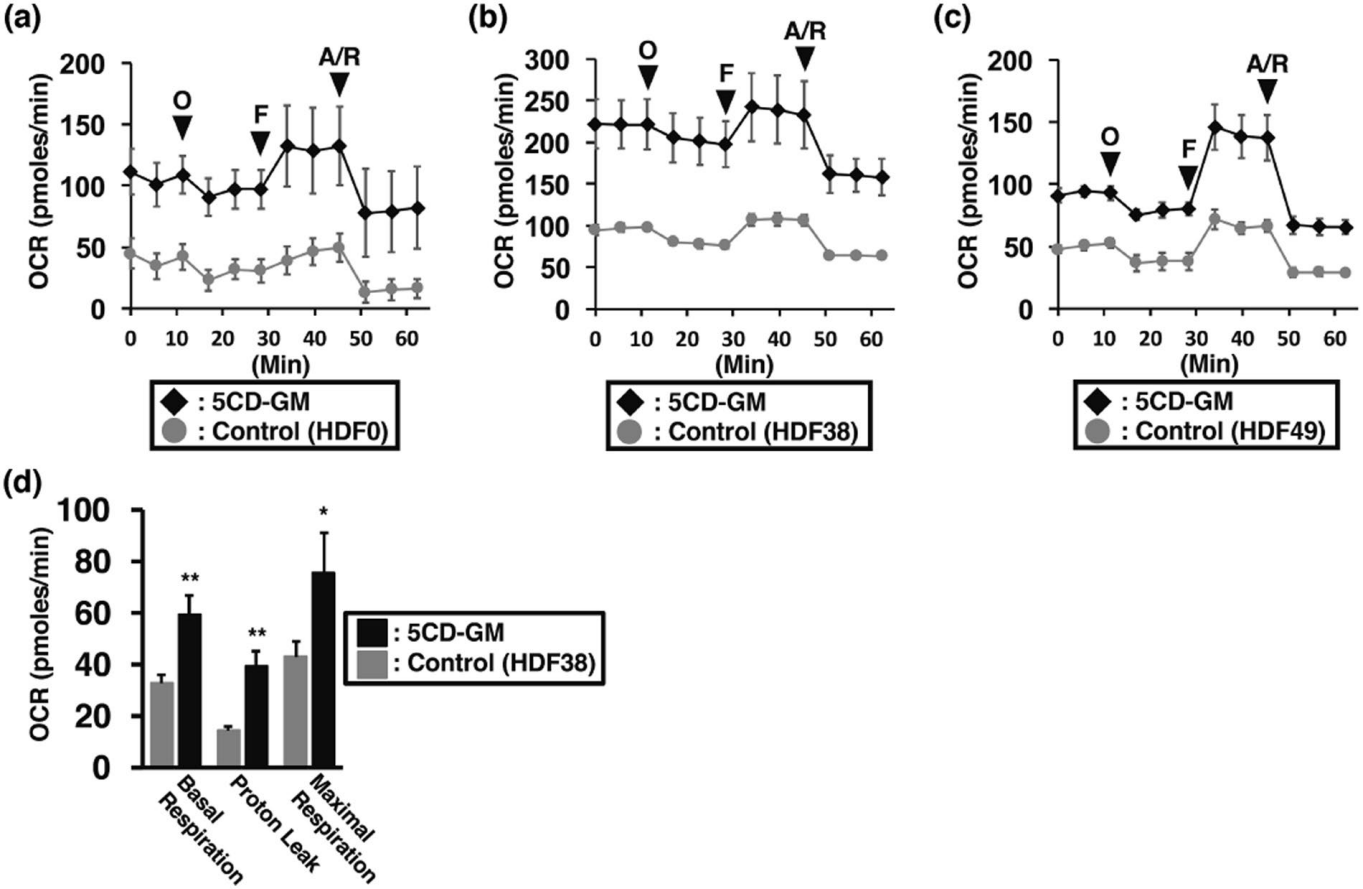
Increased oxygen consumption rate (OCR) in ciBAs. OCR was measured using the Flux analyzer in the control cells (the adipocyte medium only, grey circles) and ciBAs (black diamonds) derived from the 3 lines of human fibroblasts, HDF0 (**a**, n = 5), HDF38 (**b**, n = 3), and HDF49 (**c**, n = 4). Oligomycin (O), FCCP (F), and Antimycin A/Rotenone (A/R) were added at indicated times to final concentrations at 2, 0.25, and 0.5 μM, respectively. (**d**) The OCR for basal respiration, proton leak, and maximal respiration was compared between the control and ciBAs derived from HDF38 fibroblasts (n = 3). Data represent mean ± SD. Student’s t-test: *P < 0.05, **P < 0.01.

To demonstrate that increase of mitochondria in ciBAs is associated with higher rate of oxygen consumption, ciBAs derived from the 3 lines of human fibroblasts were analysed by Flux analyser. The values of OCR were typically varied by adding perturbation reagents during the measurement, and all the ciBAs derived from HDF0, HDF38, and HDF49 fibroblasts exhibited higher OCR compared to the control (Fig. 5a,b,c). The OCR for basal respiration, proton leak, and maximal respiration was significantly increased in the ciBAs derived from HDF38 compared to the control (Fig. 5d). In particularly, the higher OCR for proton leak indicates an increased rate of uncoupling respiration, which implies that induced UCP1 in ciBAs might be functional. These results suggest that oxidative metabolism in mitochondria is elevated in ciBAs, consistent with the observation of increased cellular mitochondrial signals in ciBAs.

## Discussion

Obesity is a major risk factor for development of various metabolic diseases including diabetes, cardiovascular diseases, and cancer. Accumulated evidence has suggested that brown adipocytes are responsible for a balance of energy metabolism and heat production, and are important for suppression of weight gain and metabolic diseases not only in the mouse but also in humans^18–20^. One of the recent findings is that the amount of brown-like adipocytes (beige adipocytes) in white adipose tissues can be increased by β-adrenergic stimulation, cold exposure, and exercise, which is associated with significant improvement of energy metabolism^21^. In addition, most commercial anti-obesity drugs are dependent on energy restriction by repression of appetite or inhibition of nutrient absorption, which carries a potential risk of adverse effects on human health^22^. More secure and healthier therapies are required to ameliorate obesity by enhancing energy expenditure. Therefore, the increase of brown adipocytes in the body is a promising strategy for prevention of obesity and related metabolic diseases. Thus, brown adipocytes have become a more attractive target to modify human metabolism.

Direct reprogramming without gene induction is a promising approach for future cell transplantation therapies and *in vivo* direct reprogramming. Overexpression of two transcription factors, C/EBP-β and C-Myc, has been recently reported to directly convert human fibroblasts into brown adipocytes^23^. However, direct reprogramming with exogenous gene induction carries a potential risk for malignant tumor formation after transplantation. In this study, we successfully developed a novel method to generate transgene-free brown adipocytes from human fibroblasts derived from different donors with the chemical cocktail. In addition, we actively optimised the combination of chemical compounds, as shown in Supplementary Figs S1–S5. Finally, we identified the optimum chemical combination, 5CD-GM (SLDF), for the direct conversion into the brown adipocyte-like cells.

**Figure 6:**
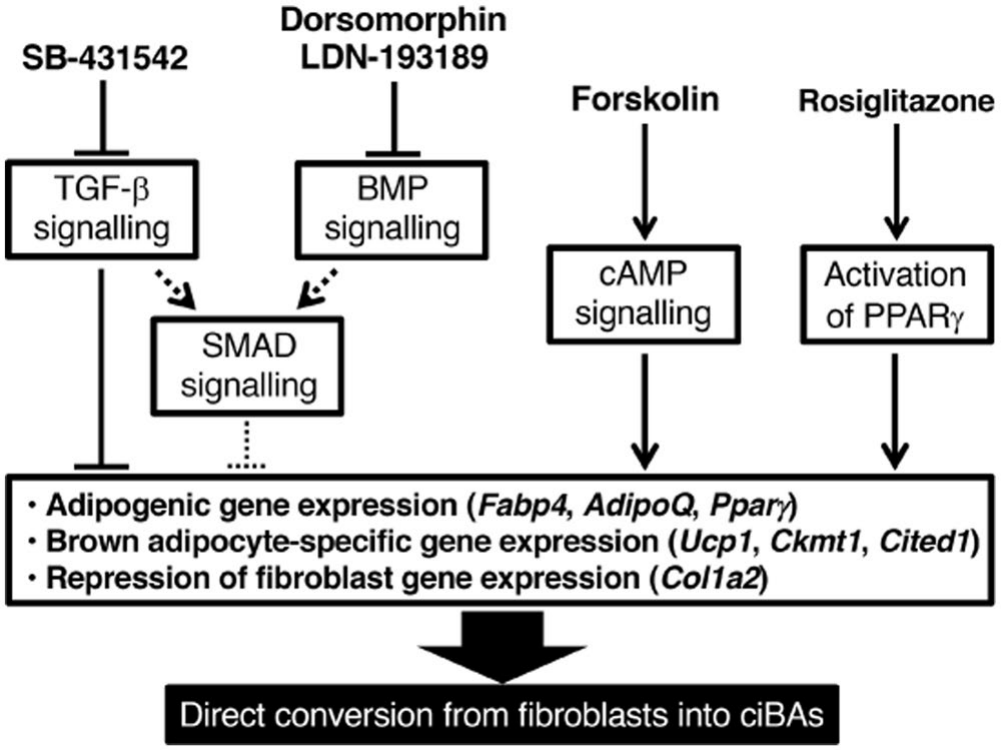
A schematic model for a possible connection between signalling pathways regulated by each chemical compound and the direct conversion into ciBAs from human dermal fibroblasts. Our results obtained using SB-431542, an inhibitor of TGF-β signalling pathway, suggest that TGF-β signalling pathway negatively regulates adipogenesis, and that the removal of SB-431542 from the chemical cocktails strongly suppressed the conversion efficiency. BMP signalling pathway regulated by the inhibitors, LDN-193189 and Dorsomorphin, is likely involved in modulation of the activity of downstream SMAD proteins shared with TGF-β signalling pathway. Cellular cAMP induction by Forskolin is closely associated with the activation of *Ucp1* and other thermogenic genes through several kinases. Rosiglitazone plays a critical role in adipogenesis for both white and brown adipocytes through PPARγ activation. The chemical combination of 5CD-GM and rosiglitazone might promote gene expression of adipogenic genes and brown adipocyte-specific genes in a coordinated manner, which might be associated with a stable conversion into ciBAs from human dermal fibroblasts.

Our study showed that CHIR99021 (G), a GSK3β inhibitor, rather inhibited adipogenesis (Fig. 1 and Fig. S1), suggesting that WNT signalling pathway negatively regulates adipogenesis or the direct conversion into brown adipocytes. Corresponding to our results obtained using SB-431542 (S), TGF-β signalling pathway also negatively regulates adipogenesis (Fig. 6) and TGF-β signalling pathways are more activated in the white adipose tissue of obese mice than lean mice^24–26^. Our experiments also showed that in most cases the treatment with SB-431542 (S), an inhibitor of TGF-β signalling pathway, promoted the generation of ciBAs and that the removal of SB-431542 from the chemical cocktails strongly suppressed the conversion efficiency (Figs S1–S5). BMP signalling pathway regulated by the inhibitors, LDN-193189 (L) and Dorsomorphin (D), is involved in modulation of the activity of downstream Smad proteins in collaboration with TGF-β signalling pathway^27^. This is likely why these inhibitors can affect the efficiency of the direct conversion into brown adipocytes. In addition, cellular cAMP induction by Forskolin (F) is closely associated with the activation of *Ucp1* and other brown adipocyte genes through several kinases^28^. Moreover, as previously reported, rosiglitazone plays a critical role in adipogenesis for both white adipose tissue (WAT) and BAT through PPARγ activation. In our experiments, the exclusion of rosiglitazone from 5CD-GM combination little generated the adipocyte-like cells (data not shown), suggesting that rosiglitazone synergistically affects the direct conversion with the other chemical compounds.

In this study, we successfully identified a novel method to convert human fibroblasts into brown adipocytes by using combinations of small compounds. However, several important points still have to be answered for future transplantation therapy and *in vivo* direct reprogramming into ciBAs. Our study revealed that ciBAs are sensitive to isoproterenol, an agonist for β adrenergic receptors, and further induced the expression of *Ucp1*. It should be noted that even 0.1 μM isoproterenol is sufficient for the induction within 6 hrs, which implies that ciBA is functional for adaptive thermogenesis. Moreover, ciBAs showed increased oxidative metabolism likely due to increased cellular mitochondria in ciBAs. Further studies are required to identify *in vivo* functions of ciBAs in lipid and glucose metabolism after transplantation into animals. Our findings are the first step to preparing brown adipocytes from human fibroblasts by using chemical compounds only, without gene induction.

## Methods

### Cell culture

Human dermal fibroblasts were purchased from DS Pharma Biomedical Co. (Osaka, Japan). The information is listed in Table 1. About 1.5 × 10^5^ cells were seeded on a 35-mm dish with high-glucose DMEM (11995–065, Gibco, MA, USA) supplemented with 10% FBS (HyClone, UT, USA) and penicillin/streptomycin (Gibco).

### Direct conversion from human fibroblasts into brown adipocyte-like cells

After reaching 80–90% confluence of each human fibroblast line, the medium was changed to start direct conversion into brown adipocyte-like cells with adipocyte medium prepared from high-glucose DMEM (043–30085, Wako, Osaka, Japan) supplemented with rosiglitazone (Wako) and MK425 (Takara Bio Inc., Shiga, Japan). MK425 contains ascorbic acid, biotin, pantothenic acid, triiodothyronine, octanoic acid, insulin, dexamethasone, IBMX (3-isobutyl-1-methylxanthine), FBS, and penicillin/streptomycin. The final concentration of rosiglitazone was set to 1 μM concentration. Small chemical compounds were purchased from Wako, except for CHIR99021 (Cayman Chemical, MI, USA), and added to the adipocyte medium at the following final concentrations: CHIR99021 (1 μM), PD0325901 (1 μM), SB-431542 (2 μM), LDN-193189 (1 μM), Pifithrin-α (5 μM), Dorsomorphin (1 μM), and Forskolin (7.5 μM). Human fibroblasts were cultured in the adipocyte medium either with or without each chemical cocktail as indicated for 3 weeks. The medium was changed every 3 days. For maturation, the cells were further cultured in the adipocyte medium only for 1 week.

### Immunostaining

Immunocytochemistry was performed as previously reported^14^. In brief, cells were fixed with 2% paraformaldehyde (Nacalai Tesque, Kyoto, Japan). After washing 3 times with PBS, the cells were incubated with PBS containing 0.1% Triton X-100 for 10 min. Then, they were blocked with PBS containing 3% skim milk for 1 hr at room temperature. The UCP1 antibody (ab10983, Abcam, Cambridge, UK) was diluted at 1/500 with the blocking solution. The cells were incubated with the antibody overnight at 4 °C. After washing 3 times with PBS, the cells were incubated with Alexa Fluor 488 donkey anti-rabbit IgG (A-21206, Invitrogen, CA, USA) for 2 hrs at room temperature. Cell nuclei were stained with DAPI solution (Dojindo, Kumamoto, Japan). All images were obtained using a fluorescence microscope (Axio Vert.A1, Carl Zeiss, Oberkochen, Germany). Mitochondria were stained by MitoTracker® Red CMXRos (Thermo Fisher Scientific, DE, USA).

### RNA isolation and qRT-PCR

To quantify gene expression, total RNA was extracted from the fibroblasts treated with each chemical cocktail using RNeasy Mini kit (Qiagen, CA, USA). As a control, the fibroblasts were cultured with the adipocyte medium only in parallel. Total RNA was also collected from ciBAs and the control cells derived from HDF38 fibroblast line after treatment with either 0.1, 1, and 10 μM isoproterenol or Forskolin for 3 hrs and 6 hrs. After reverse-transcription by ReverTra Ace® qPCR RT Master Mix with gDNA Remover (TOYOBO, Osaka, Japan), real-time PCR analysis was performed using Power SYBR Green PCR Master Mix (Applied Biosystems, MA, USA). The reactions were carried out in triplicate and under the following conditions: 10 min at 95 °C, followed by 40 cycles of 15 sec at 95 °C and 60 sec at 60 °C. All the results were normalized by *Tbp* mRNA level. All primer sequences for qRT-PCR are listed in Table S1.

### Western blotting analysis

To evaluate protein level of UCP1, proteins were extracted from ciBAs and the control cells derived from human fibroblasts with RIPA buffer [50 mM Tris–HCl (pH 8.0), 0.15 M sodium chloride, 0.5% sodium deoxycholate, 0.1% sodium dodecyl sulphate, 1% NP-40 substitute, Wako) and protease inhibitor cocktail (Wako). They were separated by 10% SDS-PAGE and transferred to a PVDF membrane (Thermo Fisher Scientific). The membranes were blocked with 5% skim milk followed by incubation with UCP1 antibody (MAB6158, R&D systems, MN, USA) or β-actin antibody (A5316, Sigma-Aldrich, MO, USA). Then the membranes were incubated with a secondary antibody conjugated with HRP (Santa Cruz Biotechnology, CA, USA). The bands were detected by Immobilon™ Western Chemiluminescent HRP Substrate (Merck Millipore, Darmstadt, Germany).

### Measurement of oxygen consumption rate

For measurement of oxygen consumption rate (OCR) by mitochondria, 3 lines of human fibroblasts, HDF0, HDF38, and HDF49, were directly converted by the chemical cocktail, 5CD-GM, on 96-well plate for 3 weeks. For maturation, these cells were further incubated with the adipocyte medium for 1 week. As a control, each fibroblast line was cultured with the adipocyte medium in parallel. Then, after 1 hr incubation at 37 °C in a non-CO2 incubator, OCR in each well containing ciBAs or control cells was analysed by XF96 Extracellular Flux Analyzer (Seahorse Bioscience Inc., MA, USA) according to the manufacturer’s instructions. During the analysis, Oligomycin, FCCP, and Antimycin A/Rotenone were added into each well via an injection apparatus to final concentration at 2, 0.25, and 0.5 μM, respectively.

## Electronic supplementary material

Supplementary Information
