# Supplementary material for: Direct conversion of human fibroblasts to brown adipocytes by small chemical compounds
Source: Sci Rep. 2017 Jun 27;7:4304. doi: 10.1038/s41598-017-04665-x (PMC5487346; doi:10.1038/s41598-017-04665-x)
Supplement: Supplementary file 1 — Supplementary Information [file 41598_2017_4665_MOESM1_ESM.pdf]

**Direct conversion of human fibroblasts to brown adipocytes by small chemical compounds.**

Yukimasa Takeda[1*], Yoshinori Harada[2*], Toshikazu Yoshikawa[1], and Ping Dai[1]

## Supplementary Information

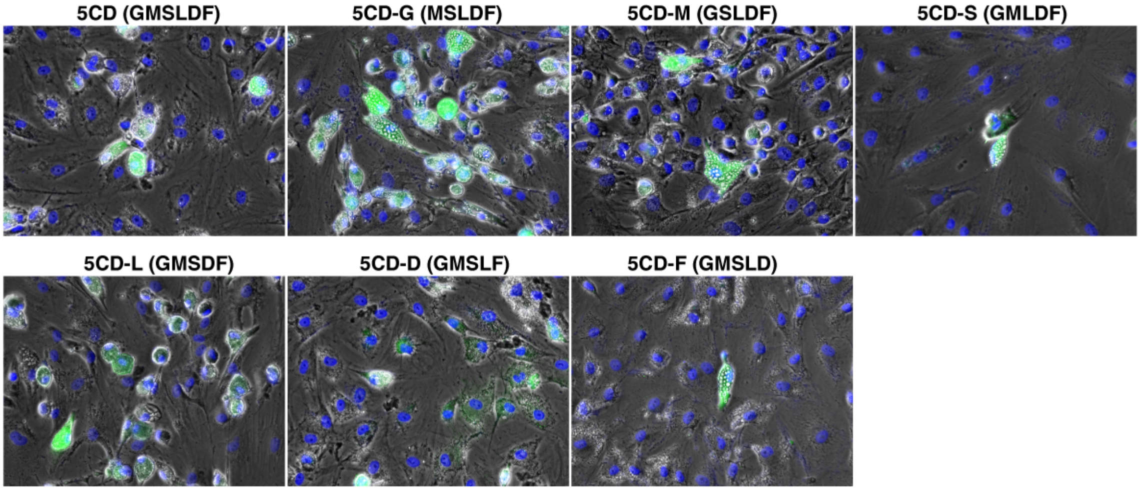


**Figure S1. Optimisation of a combination of 6 or 5 chemical compounds to directly convert human fibroblasts into brown adipocyte-like cells.**

Representative images of immunostaining for UCP1 protein (green) and nuclei (blue) in the fibroblasts (HDF38). They were cultured with each combination of 6 or 5 chemical compounds as indicated for 3 weeks followed by culturing with the adipose medium for 1 week for maturation.

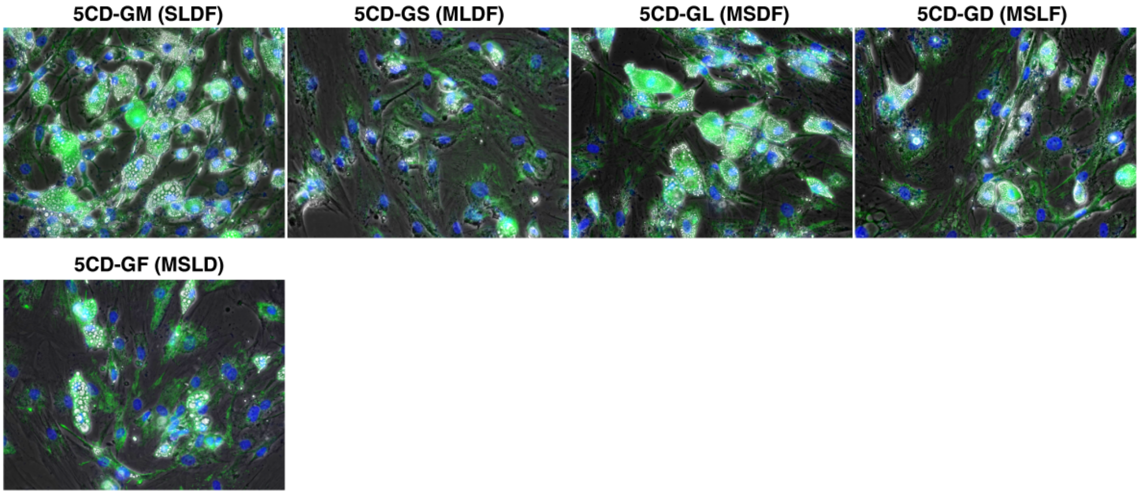


**Figure S2. Optimisation of a combination of 4 chemical compounds to directly convert human fibroblasts into brown adipocyte-like cells.**

Representative images of immunostaining for UCP1 protein (green) and nuclei (blue) in the fibroblasts (HDF38). They were cultured with each combination of 4 chemical compounds as indicated for 3 weeks followed by culturing with the adipose medium for 1 week for maturation.

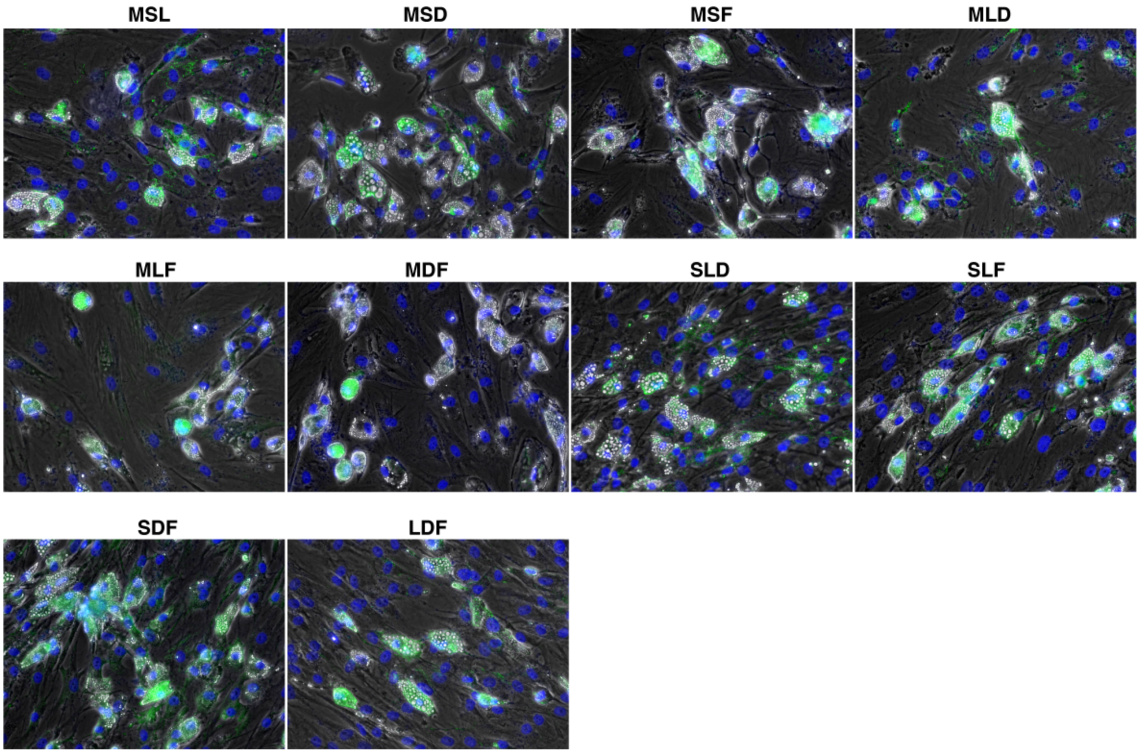


**Figure S3. Optimisation of a combination of 3 chemical compounds to directly convert human fibroblasts into brown adipocyte-like cells.**

Representative images of immunostaining for UCP1 protein (green) and nuclei (blue) in the fibroblasts (HDF38). They were cultured with each combination of 3 chemical compounds as indicated for 3 weeks followed by culturing with the adipose medium for 1 week for maturation.

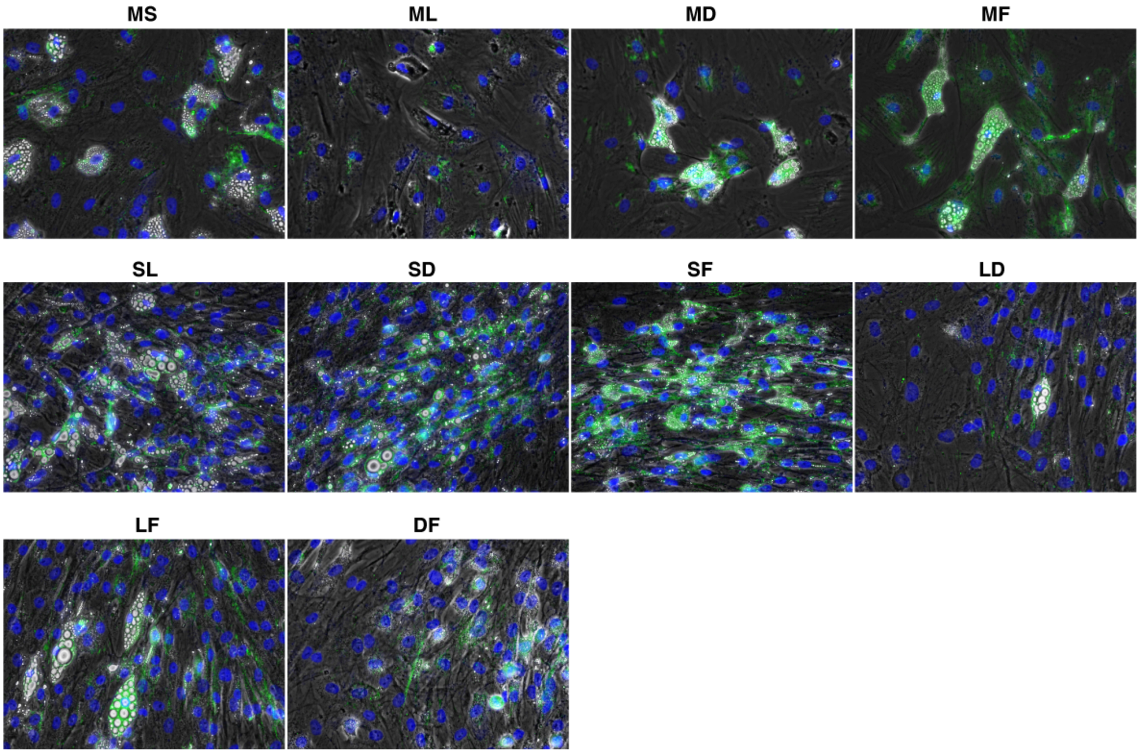


**Figure S4. Optimisation of a combination of 2 chemical compounds to directly convert human fibroblasts into brown adipocyte-like cells.**

Representative images of immunostaining for UCP1 protein (green) and nuclei (blue) in the fibroblasts (HDF38). They were cultured with each combination of 2 chemical compounds as indicated for 3 weeks followed by culturing with the adipose medium for 1 week for maturation.

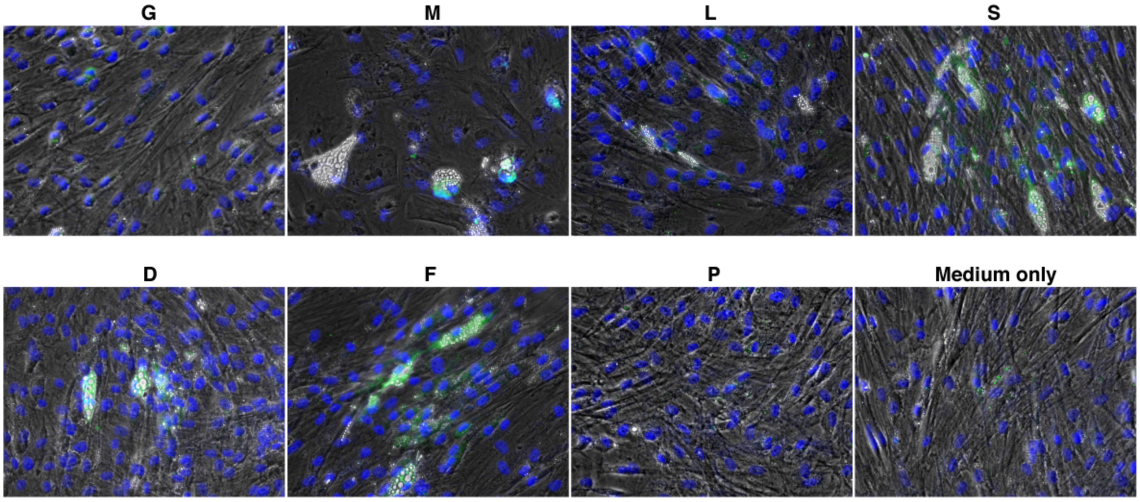


**Figure S5. Optimisation of a chemical compound to directly convert human fibroblasts into brown adipocyte-like cells.**

Representative images of immunostaining for UCP1 protein (green) and nuclei (blue) in the fibroblasts (HDF38). They were cultured with each chemical compound as indicated for 3 weeks followed by culturing with the adipose medium for 1 week for maturation.

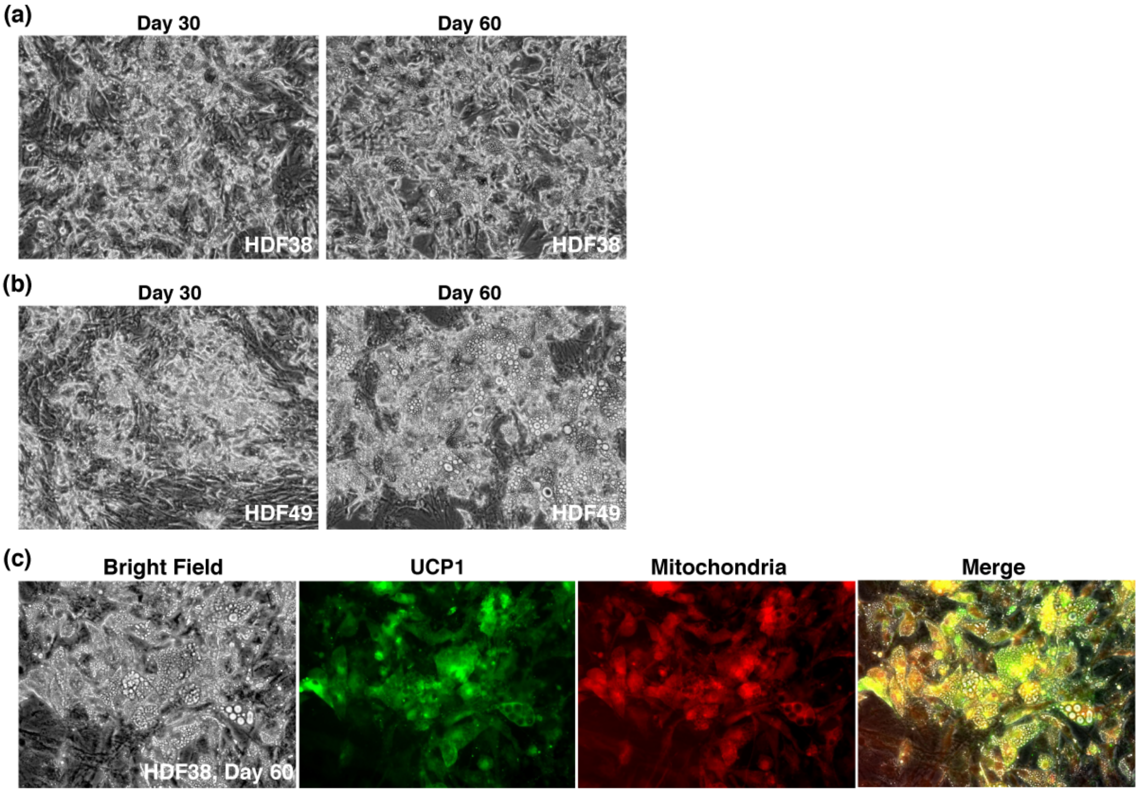


**Figure S6. The morphology and UCP1 expression of ciBAs were maintained for long-term culture.** Images of bright field in HDF38 (a) and HDF49 (b) fibroblasts at Day 30 and Day 60 after treatment with the chemical compounds, 5CD-GM, for the first 3 weeks followed by cultivation with the adipocyte medium only. (c) Images of bright field, UCP1 protein expression (green), mitochondria labelling with MitoTracker (red), and merged image of UCP1 (green), MitoTracker (red) and DAPI (blue) to visualise nuclei in ciBAs derived from HDF38 fibroblasts at Day 60.

**Table S1. Sequences of primers used in qRT-PCR.**

| Gene | Sense primer | Antisense primer |
|---|---|---|
| *Tbp* | ACTACGGGGTTATCACCTGTGAG | GTGCAGGAGTAGGCCACATTAC |
| *Ucp1* | TCTACGACACGGTCCAGGAG | GAATACTGCCACTCCTCCAGTC |
| *Ckmt1* | AGCAGGAATGGCTCGAGAC | ATCCTCCTCATTCACCCAGATC |
| *Cited1* | TGGCACCTCACCTGCGAAG | GCAGAATGGCCACTGCTTTG |
| *Col1a2* | TCGCACATGCCGTGACTTG | GATAGCATCCATAGTGCATCCTTG |
| *Fabp4* | GCCAGGAATTTGACGAAGTCA | CCCATTTCTGCACATGTACCAG |
| *AdipoQ* | CTGGTGAGAAGGGTGAGAAAG | GTTTCACCGATGTCTCCCTTAG |
| *Ppar*$\gamma$ | TGGAATTAGATGACAGCGACTTG | CTTCAATGGGCTTCACATTCAG |
